# Supplementary material for: Learning curve and surgical outcome of robotic assisted colorectal surgery with ERAS program
Source: Sci Rep. 2022 Nov 29;12:20566. doi: 10.1038/s41598-022-24665-w (PMC9709162; doi:10.1038/s41598-022-24665-w)
Supplement: Supplementary file 2 — Supplementary Information 2. [file 41598_2022_24665_MOESM2_ESM.zip › RAL Raw data-Ñ[▒K/047/OP N_ñw╝╨░O▒Kñσ.pdf]

W52 / W52

臺中榮民總醫院
手術室護理記錄

[redacted]

基本資料

- 室別： 開刀房-17室，手術科別： CRS，手術類別： 常規，日期： 2020/12/23
- 進入時間： 0700 ； 麻醉開始時間： 0717 ； Time Out時間： 0837 ； 劃刀時間： 0838 ； 手術結束時間： 1515 送出時間： 1540 ；
- 麻醉方式： GE
  診斷：rectum ca.
  手術名稱：Robot Assisted Low Anterior Resection
  手術局部用藥：NIL
  術前預防性抗生素：
  - 抗生素名稱一： Veterin 1GM IVD由麻醉護理師給予
  - 時間： 0825
  - 上次簽章: 羅媗鈴
  ，追加預防性抗生素：
  - 抗生素名稱一： Veterin 1GM IVD由麻醉護理師給予
  - 時間： 1225
  - 上次簽章: 邱櫳婇
- 手術醫師： 林俊余，楊曙榮，蔡濬樟
- 麻醉醫師： VS 陳永銘
-
  - 刷手護士A ： 邱櫳婇，時間： 0700，刷手護士B ： 陳秋誼，時間： 0700，接替刷手一： 陳映庭/陳秋誼，時間： 1230
  - 流動護士A ： 羅媗鈴，時間： 0700，接替流動一： 邱櫳婇，時間： 1200，接替流動二： 羅媗鈴，時間： 1500

皮膚狀況

A-V Shunt： 無
術前： 完整
記錄者： 上次簽章: 羅媗鈴
術後： 完整
記錄者： 上次簽章: 邱櫳婇

手術準備

手術臥式 單一手術臥式

-
  - 手術臥式： 截石臥式
  - 使用附件： 頭圈，腳架，其它： 側身架
  - 皮膚準備： 10%Aq Betadine，Hibiscrub，2% Chlorhexidine
  - 電燒： 使用電燒: 電極片位置： 右大腿，記錄者： 上次簽章: 羅媗鈴

止血帶T： 無

植入物

- 植入物名稱/號碼/數量/部位：GIA 45:45*4.3MM*4/RECTUM

，記錄者： 上次簽章: 邱櫳婇

標本送檢

- 細菌培養 無
- 細胞學 無
- 冰凍切片 無
- 病理標本
  組織名稱一：IMA root
  ，
  組織名稱二：Rectum

,
組織名稱三： Distal cut end

- 截肢 無

標本交付 標本： 1 袋 ， 標本： 0 盒 ， 標本： 2 瓶 ， 細胞學： 0 管 ， Culture Tube： 0 管 ， 被交付醫師： CR 楊曙榮 ，

- 記錄者： 羅媗鈴
- 時間： 1520

傷口術後敷料

傷口敷料：

- 請於圖上標示部位：

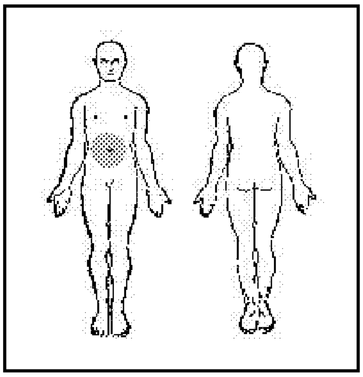

- 傷口敷料： Mepore

導管

導管	天數	導管資訊	插入日期	插入時間	更換日期	拔除日期	拔除時間	拔除原因	預計拔除/更換	預計拔除/更換日期
Penrose Drain(1)	1	號碼:12 mm.; 部位:骨盆腔;	2020/12/23	1400						
Double Lumen CVC(1)	1	號碼:8 Fr; 深度:15; 部位:jugular (右); 象限:RU;	2020/12/23	0815						
Epidural PCA(1)	1	部位:脊髓;	2020/12/23	0715						
A-line (1)	1	號碼:20 Ga; 部位:左手;	2020/12/23	0710						
Sump NG Tube(1)	1	號碼:16 Fr.; 部位:胃;	2020/12/23	0820						
Chemo Port(1)	86	部位:左胸; 象限:LU;	2020/09/29	1100						

用物計數

- 手術中出血量： 少 c.c.
- 手術中尿量： 200 c.c.
-
    - 其它： 無

用物計數： 多切口同時縫合

- 1.器械： 正確 ， 2.縫針： 正確 ， 3.刀片： 正確 ， 4.紗布： 正確 ， 5.腹部墊： 未使用 ， 6.紗球： 未使用 ， 7.PEANUTS ： 未使用 ， 8.紗條： 未使用 ， 9.絲帶： 正確 ， 10.針頭： 未使用 ， 11.Vessel loop： 未使用 ， 12.Boots： 未使用 ， 13.Jelco： 未使用 ， 14.棉條： 未使用 ，
    - 第一次：
        - 刷手護士 ： 陳映庭
        - 流動護士： 上次簽章: 邱櫂婇
        - 時間： 1410

,
    - 第二次：
        - 刷手護士 ： 陳映庭
        - 流動護士： 上次簽章: 邱櫂婇
        - 時間： 1415

,

- 第三次：
  - 刷手護士 ： 陳映庭
  - 流動護士： 上次簽章: 邱欐綵
  - 時間： 1420

備註

備註：XI內視鏡0度鏡 NO.1，30度鏡NO.2

- 記錄者： 上次簽章: 羅媗鈴
- 時間： 0900

填寫護理人員 羅媗鈴

正式摘要

W52 / W52

doctorRank:B
